# Smart Fault-Detection Machine for Ball-Bearing System with Chaotic Mapping Strategy

**DOI:** 10.3390/s19092178

**Published:** 2019-05-10

**Authors:** Shih-Yu Li, Kai-Ren Gu

**Affiliations:** 1 Graduate Institute of Manufacturing Technology, National Taipei University of Technology, Taipei 10608, Taiwan; 2 Department of Mechanical and Mechatronic Engineering, National Taipei University of Technology, Taipei 10608, Taiwan; t107408042@ntut.org.tw

**Keywords:** feature production, chaotic mapping, smart machine, non-destructive sensing, machine learning

## Abstract

In this paper, a set of smart fault-detection approach with chaotic mapping strategy is developed for an industrial ball-bearing system. There are four main statuses in this ball-bearing system: normal, inner race fault, outer race fault, and ball fault. However, it is hard to simply classify each of them through their vibration signals in time-series. By developing a nonlinear error dynamic system as well as a chaotic mapping strategy, the signals in the time series can be converted into the chaotic domain, which are revealed in 3D phase portraits. Further, through collocation of clustering methods, such as Euclidean distance (ED) and the kernel method of K-means (KM), the proposed 3D phase portraits of each different state can be efficiently identified through checking the autonomously adjusted ranges of feature values. The experiment results show that the proposed smart detection approach is effective and feasible, and the accuracy of detection in the testing stage is close to 100%.

## 1. Introduction

In the process of modern smart industrial manufacturing, rotating machines and particularly induction motors present an important role in industrial systems and are probably one of the most critical equipment in many industrial applications such as automatic factory, aerospace, chemical, and domestic appliances industries. There is increasing demand for real-time monitoring and detection in these industries, in which the performance of rotating machines goes beyond facilitating the advanced arrangement of maintenance schedules. These rotating machines are composed of several elements such as stator, rotor, shaft, and bearings. The bearings are the most important mechanical elements of rotating machinery. They are employed to guide and support the shafts in rotating machinery. For example, common erratic operations cause bearing failure of rotating machines, Therefore, any fault in the bearings can lead to losses on the level of production and equipment as well as potentially unsafe, ultimately a waste of time, manpower, and money.

Common ball bearings for rotating machines are made up of four parts: the outer race, the inner race, the ball, and the ball cage. In a rotating machine using the ball bearing, the ball is located between the outer race and the inner race, and the ball is in contact with the inner race, which helps to reduce the friction caused by rolling to the rotating shaft. However, even if the bearing is operating in a normal working environment, it must withstand a number of factors, such as repeated lateral stress leading to fatigue wear, ambient climate temperature difference and humidity that cause rust and may eventually make the machine become inoperable, and the fault structure, as according to the composition of the bearing, the fault state can be divided into outer race fault, inner race fault, ball fault, and cage fault.

For these reasons, condition monitoring and fault detection of these bearings have become a fundamental axis of development and industrial research. According to reliable data, about 50 percent of the rotating machinery faults are caused by the failure of the rolling bearings [1,2,3,4,5]. The mechanical vibration signal [6,7,8,9] is one of the most effective and rich sources of information for understanding phenomena related to bearing defects. The traditional fault diagnosis method is artificial feature extraction, which then carries out feature selection, dimension reduction, and classifier design [10,11,12]. Feature extraction is the most important step in the traditional method. The commonly used methods include wavelet analysis [13,14,15,16], empirical mode decomposition [17,18,19], and Hilbert spectrum [20,21,22]. However, the above methods require manual design features and are difficult to use in practical applications. Moreover, there are also several techniques that can be employed to detect and diagnose the bearing faults like Fast Fourier Transform (FFT) [23,24,25] and Short Time Fourier Transform (STFT) [26,27,28]. Those methods have a good fault condition recognition rate, but one of the problems associated with the STFT is the use of a fixed window for the entire signal, which allows obtaining a fixed resolution. Moreover, the limitation between time and frequency resolutions means that good resolution in both time and frequency cannot be achieved at the same time. On the other hand, artificial neural network, especially for the deep learning structure, has obtained huge success in the identification field, such as Coevolution Neural Network (CNN), Recurrent Neural Networks (RNN), etc. [29,30,31,32,33]. However, large amounts of training data, supervised learning requirement, and the machine with large as well as intensive computing powers are necessary. Further, an alternative approach, chaos synchronization error dynamics with fractal theory, is proposed for fault detections [34,35,36], whereby the characteristics are intercepted by fractal theory [37,38].

According to the descriptions mentioned above, for those traditional methods, high accuracy rate, efficiency, and real-time monitoring with less computational resources cannot be obtained at the same time. In addition, less amounts of data are considered in the detection approaches, which make it incapable of resolving unexpected machine problems and disturbances. As a consequence, in this article, a smart fault-detection machine is developed, which attempts to propose a set of strategies with simple, less computational resources and high accuracy and efficiency. By developing a nonlinear error dynamic system as well as a chaotic mapping strategy, the signals in the time domain can be converted into the chaotic domain to further produce the related key features in 3D phase portraits. Further, through appropriate extractions of feature values, such as Euclidean distance feature value (EDFV) and K-means feature values (KMFV), the four different states of the ball-bearings can be efficiently identified through checking the ranges of feature values automatically. More importantly, the ranges of the feature values for the four states can be autonomously adjusted via the developed algorithm. Moreover, in order to provide a more applicable strategy, 20,000 data are designed to be the training as well as testing unit, which represents around 12.5 revolutions, and spends only 0.4 s, and can then remove or reduce any disturbances and unexpected problems. This proposed smart machine attempts to provide a simpler, efficient, as well as feasible strategy for real-time monitoring of ball-bearing systems. 

The organization of this paper is described as follows. In Section 2, a complete flowchart of the smart machine for fault detection of the ball-bearing system is introduced step by step. In Section 3, three different testing strategies are provided, and the training as well as testing results are investigated. Finally, a conclusion is provided in Section 4.

## 2. Materials and Methods

**Figure 1:**
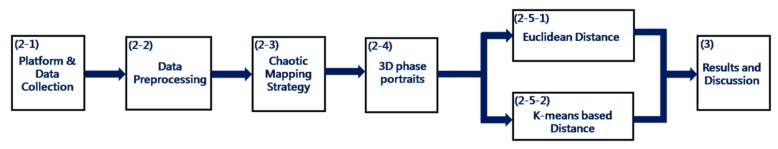
The flowchart of the proposed smart fault detection machine.

In this section, a complete flowchart of this proposed smart machine for fault detection of a ball-bearing system is introduced in detail, which has been organized in Figure 1. In Section 2.1, the information about the experimental platform and the collected data of different kinds of states are revealed. Section 2.2 describes the preprocess of the collected data, while Section 2.3 discusses the strategy of chaotic mapping and the applied dynamic systems. The four different states, comprising normal, inner race fault, outer race fault, and ball fault, are transformed into the chaotic domain, which represents their key features via 3D phase portraits in Section 2.4. Finally, several feature detection methods are constructed in Section 2.5. The developed smart machine for fault detection of a ball-bearing system has been investigated via applying the data from the testing set, which are discussed in Section 3.

### 2.1. The Experimental Platform and Data Collection

**Figure 2:**
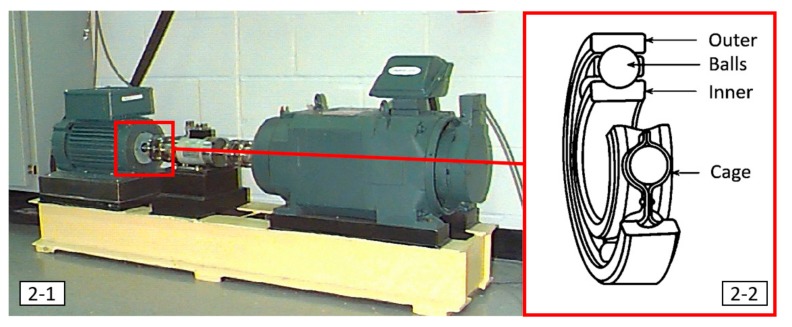
The introduction of experimental platform for the ball-bearing system.

In this sub-section, the complete information of the experimental platform is presented, and the collected data of different kinds of states are revealed. All the data sources are delivered from the U.S. Case Western Reserve University-Bearing Data Center [39]. The experimental platform of the ball-bearing system is shown in Figure 2, where the dynamometer and control electronics are arranged at the right-hand side, and a 2 Hp motor, torque transducer/encoder, and motor spindle for supporting the bearing are designed at the left-hand side. The kernel structure of the ball-bearing system is further highlighted in Figure 2-2, which shows there are four main parts in this system: outer race, inner race, balls, and the cage encasing the balls for fixing the balls. 

Experiments were executed using a 2 HP Reliance Electric motor for data collection. The vibration data was collected using accelerometers, digital data was collected at 12 KHz, and data was also collected at 48 KHz for drive end bearing faults, wherein the accelerometers were placed at the 12 o’clock position at both the drive end and fan end of the motor housing. Interestingly, the outer race faults are stationary faults, and thus placement of the fault relative to the load area of the bearing has a direct impact on the vibration response of the motor/bearing system. In order to quantify this effect, experiments were executed for both fan and drive end bearings with outer race faults situated at 12 o’clock, at 6 o’clock (vertical to the load area), and at 3 o’clock (straight in the load area). Simultaneously, speed (rpm) and load (HP) data were collected using the torque transducer/encoder and were recorded by hand. Finally, the acquired data were processed using the Matlab software, and generated all data files are in Matlab (*.mat) format.

The complete information of the data collected via the proposed experimental platform is summarized in Table 1, where the sampling rate are designed to be 48 K(Hz), and the laboratory provides the normal and fault ball bearing test data, and uses EDM to embed fault conditions in diameters of 7 mil, 14 mil, and 21 mil and in a depth of 0.011 inch in the outer race, inner race, and balls of the bearing. Further, four motor loads, 0–3 horsepower (HP), are considered in the proposed ball-bearing system; therefore, different kinds of motor speeds as well as their corresponding sampling points in 1 revolution are also presented in Table 1. This table provides a clear direction for researchers to analyze and process the related data through this platform.

### 2.2. Data Preprocessing 

In this sub-section, the preprocess of the collected data provided via the data center of the U.S. Case Western Reserve University are introduced. The detailed conditions of the data applied in this article are presented in Table 2, where the sampling rate is 48 KHz, the motor load is considered to be 0 HP, motor speed is set to be 1797 (rpm), which represents 1 period of motion (1 revolution) and should be around 0.0339 secs. Moreover, according to the 48 KHz sampling rate, 1602 sampling points can be obtained within 1 period of motion. Finally, the data center [39] uses EDM to embed fault conditions in diameters of 7 mil, 14 mil, and 21 mil and in a depth of 0.011 inch in the outer race, inner race raceways, and balls of the bearing, respectively, and three main different fault diameters should be investigated. The total data of the ball-bearing system for each of the states is around 240,000.

**Figure 3:**
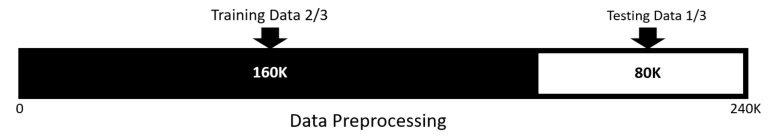
The design of training data set and testing data set.

In order to design as well as investigate the autonomous smart fault-detection machine for the ball-bearing system, the proposed total data of each states are divided into two parts, the first two-thirds of the data is set to be the training set, and the last one-third of the data is applied to be the testing set, which is shown in Figure 3. Consequently, if the total numbers of the original data are around 240,000, then the numbers of training data set are around 160,000, and the numbers of testing data set are around 80,000. 

In addition, it is worth mentioning that there are many research achievements focusing on fault detection of ball-bearing systems with one revolution, which means around 1602 data are applied to the fault detection. However, in real industrial applications, disturbance as well as unexpected machine problems occur often, therefore, more revolutions but not large amounts of data are capable to provide more convinced detection results, and those disturbances and unexpected problems can be removed or reduced as well. In this study, 20,000 data are designed to be a training as well as testing unit, which represents around 12.5 revolutions, and spends only 0.4 s. Through these designs, an effective and precise smart machine for fault detection can be developed.

### 2.3. Chaotic Mapping Strategy

In this sub-section, the complete process of the chaotic mapping strategy is presented, where the original vibration signals are mathematically mapped into the chaotic domain via applying nonlinear error dynamic systems. In this research work, the nonlinear error dynamic systems are built up via two identical chaotic systems, where the first one is the main system, and the other one is designed to be the data-feeding system. Initial conditions are given into the proposed main system for numerical iterations, where the parameters of the main systems are set to be the parameters which are able to inspire the dynamic system to reveal chaotic behaviors. For the data-feeding system, the collected data provided via the center are fed into this system technically.

**Figure 4:**
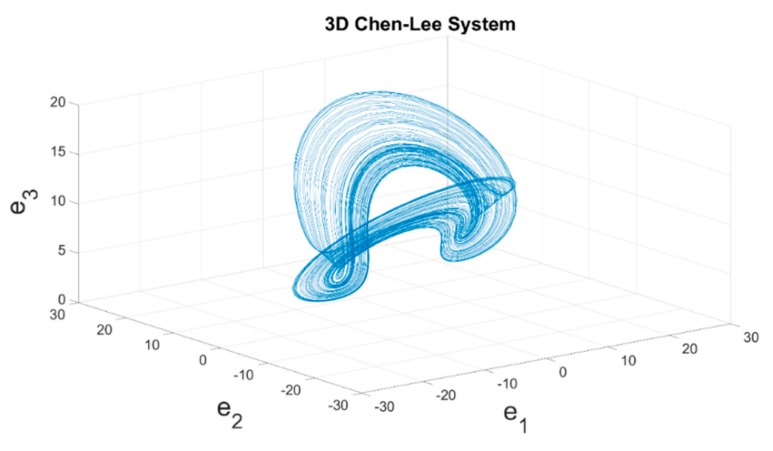
3D phase portraits of the Chen–Lee System.

As a consequence, one of the well-known nonlinear dynamic systems, the Chen–Lee System [40], is introduced to be the main system, which is described in Equation (1):

(1) $$\left\{\begin{array}{l} {\dot{x}}_{1}=-x_{2}x_{3}+ax_{1}\\ {\dot{x}}_{2}=x_{1}x_{3}+bx_{2}\\ {\dot{x}}_{3}=\left(\frac{1}{3}\right)x_{1}x_{2}+cx_{3} \end{array}\right.$$

where *x*(*t*) = [*x*_1_(*t*), *x*_2_(*t*), *x*_3_(*t*)] refers to the system states of the main system; *a*, *b*, and *c* are system parameters. Chaotic behaviors can be inspired via setting the initial conditions as (*x*_10_(*t*), *x*_20_(*t*), *x*_30_(*t*)) = (0.01, 0.02, 0.03), and system parameters *a =* 5, *b =* −10, *c* = −3.8. The three-dimensional (3D) phase portraits of chaotic trajectories are shown in Figure 4.

For the data-feeding system, an identical Chen–Lee System is also utilized, where the system parameters are set to be the same as the parameters in the main system. The data-feeding system can be concluded as follows in Equation (2), where *y*(*t*) = [*y*_1_(*t*), *y*_2_(*t*), *y*_3_(*t*)] represents the system states of data-feeding system.

(2) $$\left\{\begin{array}{l} {\dot{y}}_{1}=-y_{2}y_{3}+ay_{1}\\ {\dot{y}}_{2}=y_{1}y_{3}+by_{2}\\ {\dot{y}}_{3}=\left(\frac{1}{3}\right)y_{1}y_{2}+cy_{3} \end{array}\right.$$

The obvious differences between main system and data-feeding system are the way to input the data, and the strategy to output the results. In the main system, Equation (1), appropriate initial conditions are set to be the initial system input, and then the proposed main system provides system output through mathematical iterations, continuously. However, the mission of the data- feeding system is to feed those data in technically, i.e., the vibration signals for each of different four states should be fed into Equation (2). As a result, the original one-dimensional vibration signals (time series) are reconstructed to be three-dimensional signals via applying the concept of phase space reconstruction, which can be described as follows:

(3) $$\left\{\begin{array}{l} y_{1}=\mathrm{Data}\left[i\right]\\ y_{2}=\mathrm{Data}\left[i+1\right]\\ y_{3}=\mathrm{Data}\left[i+2\right] \end{array}\right.$$

where Data refers to the original vibration signals, *i*, *i+*1, *i+*2, reveal the data reconstructions are the time-delay of the original one, and *i* represents the beginning of the data feed into the data-feeding system. It is worth to mention that the data feeding of Equation (3) should be one unit, which means 20,000 sampling data are reconstructed via Equation (3), and inputted into Equation (2) each time, for training stage as well as for testing stage. 

Finally, the error states are designed to be *e*(*t*) = [*e*_1_(*t*), *e*_2_(*t*), *e*_3_(*t*)], where *e*_1_ = *x*_1_ − *y*_1_*, e*_2_ = *x*_2_ − *y*_2_, *e*_3_ = *x*_3_ − *y*_3_. Then, the chaotic error dynamic system can be obtained to be ${\dot{e}}_{1}={\dot{x}}_{1}-{\dot{y}}_{1}$, ${\dot{e}}_{2}={\dot{x}}_{2}-{\dot{y}}_{2}$, and ${\dot{e}}_{3}={\dot{x}}_{3}-{\dot{y}}_{3}$. Through the proposed chaotic mapping strategy with constructed nonlinear error dynamic system, the original data can be mathematically mapped into the chaotic domain; any small difference from vibration signals in each kind of state can be enlarged and revealed to be the key features.

### 2.4. Three-Dimension Phase Portraits—Feature Productions

The 3D phase portraits of those converted vibration signals through chaotic mapping strategy are developed in this Section 2.4, where key features can be obtained and further extracted via several methods. First of all, in the training stage for each of the different states, a unit (200,00 data) in the considered training set is randomly picked up 1000 times, which means that in total 20,000,000 data have been inputted to the proposed smart machine for extraction of key features. Further, there are four different states, therefore, 80,000,000 data should be inputted to the smart machine in the training stage.

**Figure 5:**
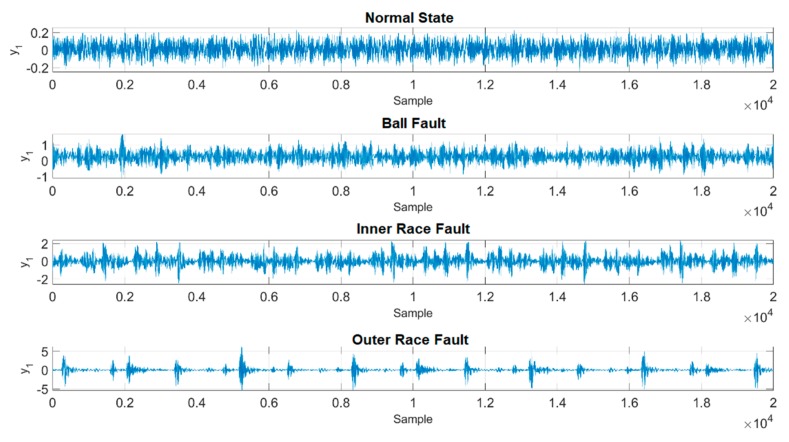
Time series of original vibration data (y).

**Figure 6:**
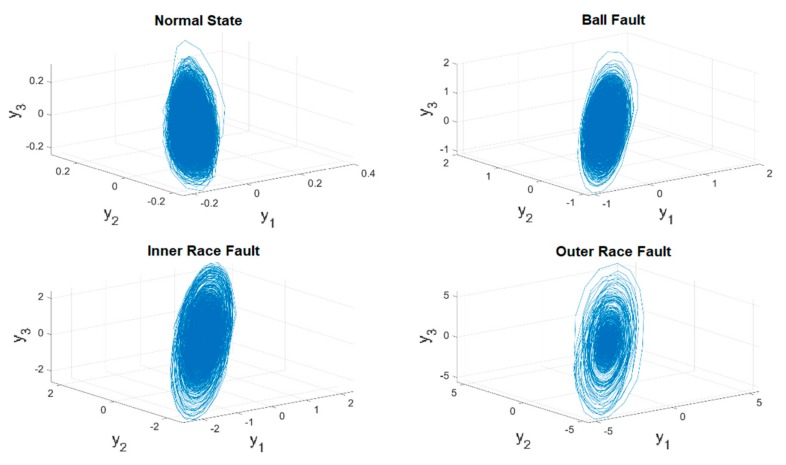
3D phase portraits of original vibration data (y).

In this case, one of the time series of original vibration signals as well as their 3D phase portraits in one training unit are provided in Figure 5 and Figure 6. However, it is hard to classify each of them through observation, and in addition, the ranges and the shapes of the proposed figures are very close to each other. As a result, a chaotic mapping strategy is presented to further address this issue. 

**Figure 7:**
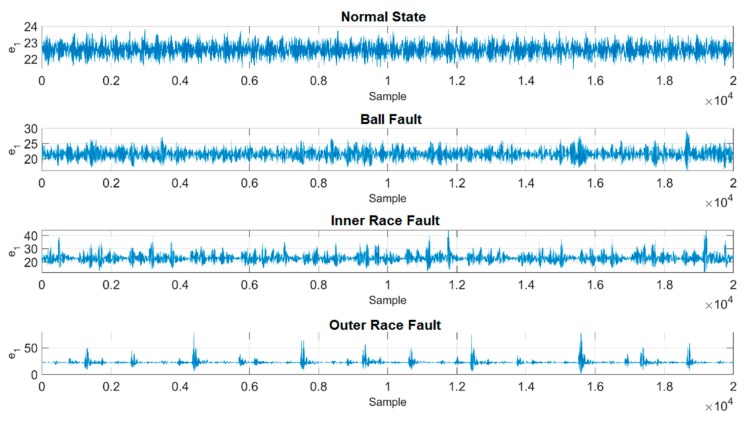
Time series of converted data in the chaotic domain.

**Figure 8:**
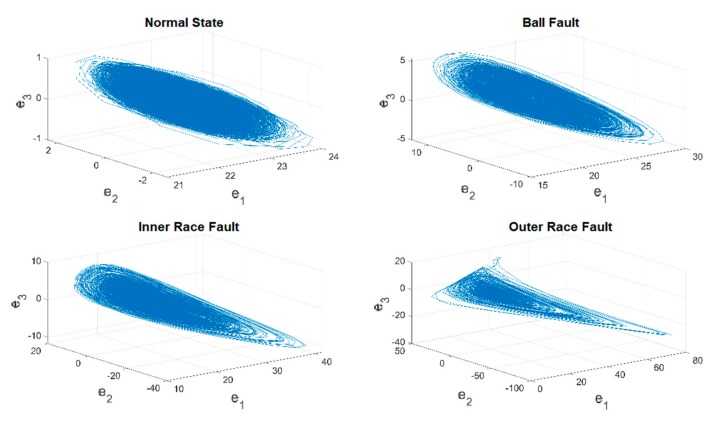
3D phase portraits of converted data in the chaotic domain.

The time series of the original vibration signals in Figure 5 are fed into the chaotic error dynamic system mentioned in the previous Section 2.3, i.e., the original signals are mapped into the chaotic domain mathematically. Figure 7 and Figure 8 show the complete time histories as well as 3D phase portraits of the four different states. In Figure 7 and Figure 8, the range of each state is distinct from the original one, the difference of range has been enlarged to be distinguishable. Moreover, in Figure 8, the shapes of the 3D phase portraits are significantly different from each other, and can be simply determined via visual recognition. Appropriate as well as unique features for the different states have consequently been produced. Eventually, the hierarchical feature extraction approach is constructed, the Euclidean distance is applied to further extract the key features, called the Euclidean distance feature value (EDFV), and K-means Clustering feature value (KMFV) is utilized to recognize the key features via using K-means Clustering as well.

### 2.5. Procedure of Feature Extraction

#### 2.5.1. Feature Extraction of Smart Machine—Euclidean Distance

**Figure 9:**
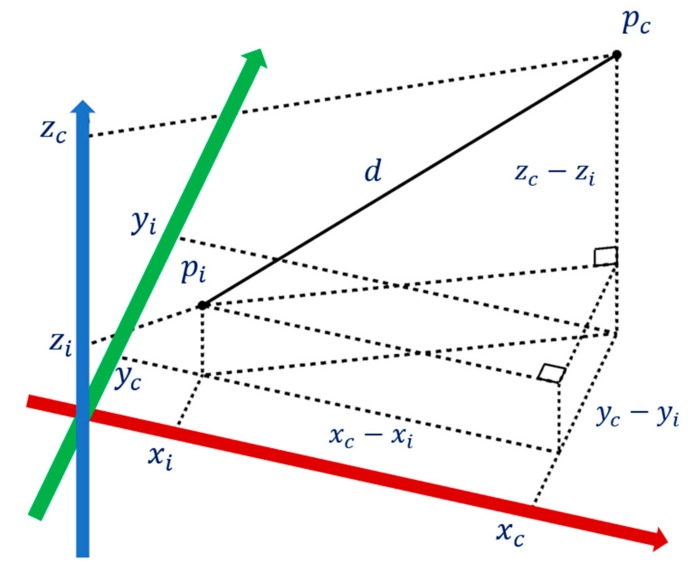
The schematic diagram of 3D Euclidean Distance.

The Euclidean metric (and distance magnitude) is that which corresponds to everyday experience and perceptions, which is one of the most common features used in machine learning. In the proposed strategy, for each different state, the average Euclidean Distances from each point *p_i_* to the central point *p_c_* in the 3D phase portraits are calculated to be the key features, which are named as Euclidean Distance features values (EDFV), the schematic diagram is shown in Figure 9. The extractions process can be organized below:

For each of different state:

**Step 1.**  Calculate the central point of the 3D phase portraits *p_c_*;
**Step 2.**  Obtain the Euclidean Distances from each point *p_i_* to *p_c_* via applying Equation (4);
**Step 3.**  Get the average of Euclidean Distances, EDFV;
**Step 4.**  There are 20000 points in the proposed 3D phase portraits for each different state, therefore, in Equation (4), *n* = 20,000.

(4) $$d_{i}=\sqrt{{\left(x_{c}-x_{i}\right)}^{2}+{\left(y_{c}-y_{i}\right)}^{2}+{\left(z_{c}-z_{i}\right)}^{2}}\;EDFV=\sum_{i=1}^{n}d_{i}/n$$

#### 2.5.2. Feature Extraction of Smart Machine—The Kernel Method of K-Means

K-means algorithm is a method of vector quantization, and now it is more popular in the field of data exploration as a clustering method, which belongs to unsupervised learning in mechanical learning, and clustering is a method of classifying data into clusters. Training data has no predefined tags. K-means clustering is a method commonly used to automatically partition a data set into k groups. It proceeds by selecting k initial cluster centers and then iteratively refining them. In this article, K is set to be one cluster to further calculate the key feature values of different four states, the concept is shown in Equation (5), and the related operation steps in this article are provided as follows:

(5) $$\arg\min_{s}\sum_{c=1}^{k}\sum_{x_{i}\in S_{c}}\|x_{i}-u_{c}{\|}^{2}$$

**Step 1.**  K is set to be one cluster, and repeat Steps 2 and 3 until the group center is fixed;
**Step 2.**  Generate a new partition by assigning each pattern to its closest cluster center;
**Step 3.**  Compute new cluster centers;
**Step 4.**  Calculate the square of distances between each point $x_{i}$ to the group center $u_{c}$;
**Step 5.**  Average all the square of distances, then KMFV can be obtained.

## 3. Results and Discussion

In this section, the training as well as testing results of the developed smart fault-detection machine is investigated, and the detailed operation procedure for the four different states are arranged in Table 3. In observation of Table 3, the total numbers of data for each of the four states, normal state, ball fault, inner race fault, and the outer race fault, are 243,938, 243,938, 244,339, 246,342, respectively, which are all around 240,000. According to the data preprocess discussed in Section 2.2, the first two-thirds of the data is set to be the training set, and the last one-third of the data are applied to be the testing set. Therefore, those data for training set as well as testing set are listed in the second and third lines in Table 3. Furthermore, 20,000 data are designed to be a training unit as well as testing unit (around 12.5 revolutions, 0.4 s), which are capable to provide more convincing detection results, meaning removed or reduced disturbances and unexpected problems. Finally, in the training stage, for each of the four states, 1000 units in the training set are randomly picked up for chaotic mapping, where Euclidean distance (ED), the kernel method of K-means (KM), and hierarchical approaches are applied to further extract key features. In the testing stage, for each of the four states, 1000 units in the testing set are randomly picked up for smart fault-detection.

**Figure 10:**
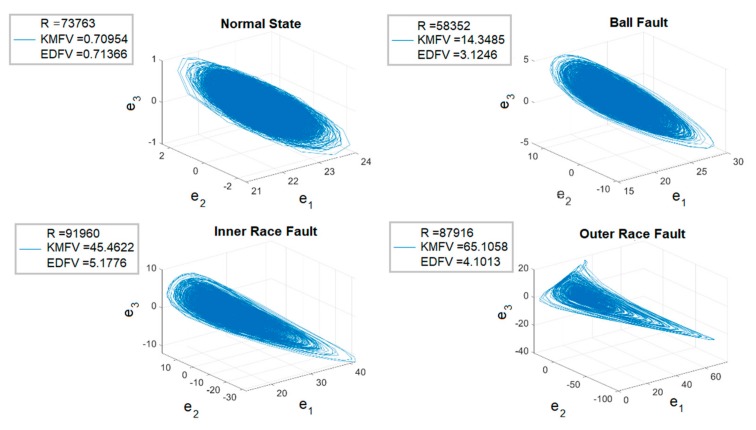
The experimental results for the training stage—3D phase portraits. KMFV, K-means-based feature value; EDFV, Euclidean distance feature value.

**Figure 11:**
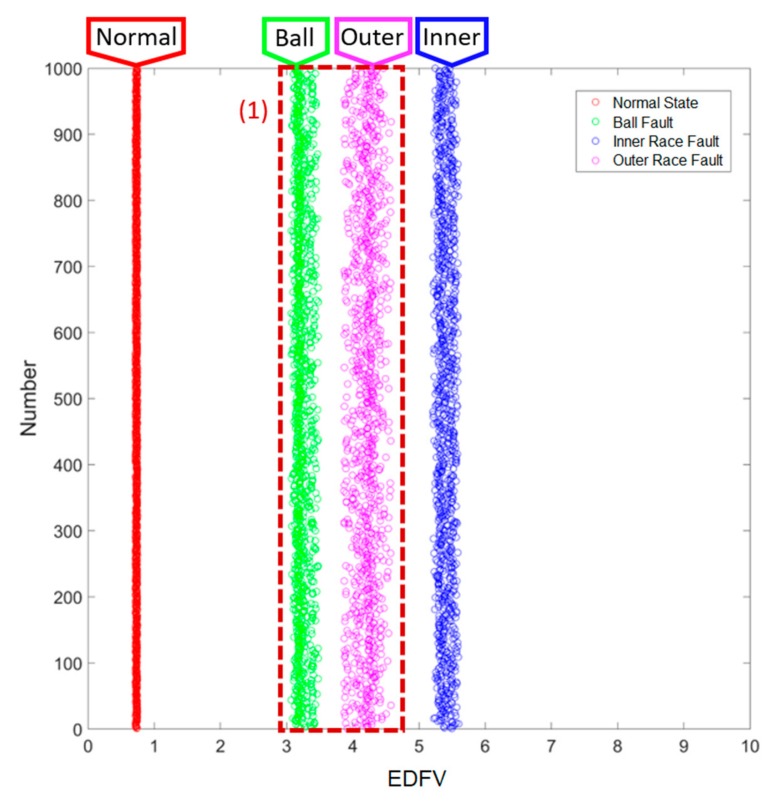
Results of Method 1: EDFV.

Following the introduction of the detailed operation procedure, 80,000 3D phase portraits of the four different states for ball-bearings can be obtained in the training stage, where Figure 10 represents one of the experimental results. In Figure 11, the four different states of ball-bearings are shown in the chaotic domain, which can be clearly identified by vision. Further, R refers to the random starting point of the training unit each time, KMFV shows the K-means-based feature value, and the EDFV represents the feature value accounted through Euclidean distance. Through extracting the related feature values from KMFV and EDFV, and autonomously adapting the range of feature values in the training stage, a complete range of feature values for different kinds of states can be concluded in Table 4.

In observation of Table 4, it is clear that each of the different states can be simply classified via the defined range of feature values, and most importantly, the ranges for the four different states can be autonomously obtained without any range overlap. In the following testing stage, three different testing strategies are provided for further study.

### 3.1. Results of Smart Fault Detection Machine—Method 1: EDFV

For testing Method 1, the range of EDFV is applied, where the last one-third of the data in the testing set are randomly picked up 1000 times in each of the different states, and the corresponding key feature values are accounted in real-time via the designed smart machine. The testing results are given in Figure 11; the horizontal axis refers to the range of key feature values, and the vertical axis reveals the testing time, 1000, for each of the different states. The four different states can be identified immediately. For the same states, the EDFV extracted via the testing data are all grouped together within close ranges, and further, for the different states, the EDFV extracted via the testing data are totally distinct. In fact, even if the current results are correct, the ranges of key feature values between the ball fault and the outer race fault are very close to each other (marked as (1)), which might lead to potential misdiagnosis. As a result, a hierarchical structure combining with EDFV as well as KMFV is developed in Section 3.2. 

### 3.2. Results of Smart Fault Detection Machine—Method 2: EDFV + KMFV (Hierarchical Structure)

**Figure 12:**
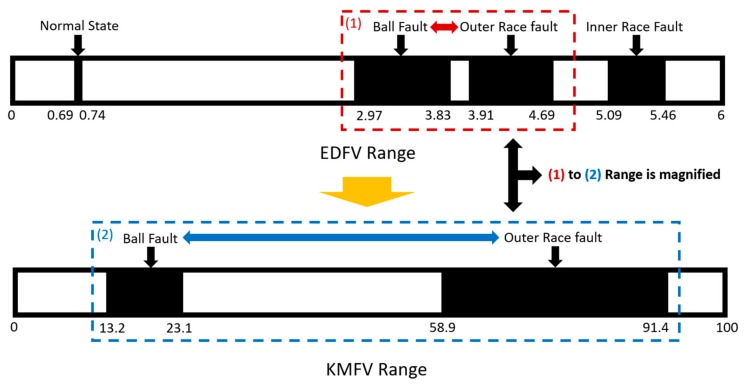
The schematic diagram of the hierarchical structure in Method 2.

For testing Method 2, the ranges of EDFV as well as KMFV are applied, which is a hierarchical strategy to further classify the different states of ball-bearings. The schematic diagram of the hierarchical structure can be summarized in Figure 12, which represents that in the first level, EDFV is applied to recognize the data to be the normal state, inner race fault state, and so on. In the second level, KMFV is applied to further identify the ball fault state and outer race state. 

**Figure 13:**
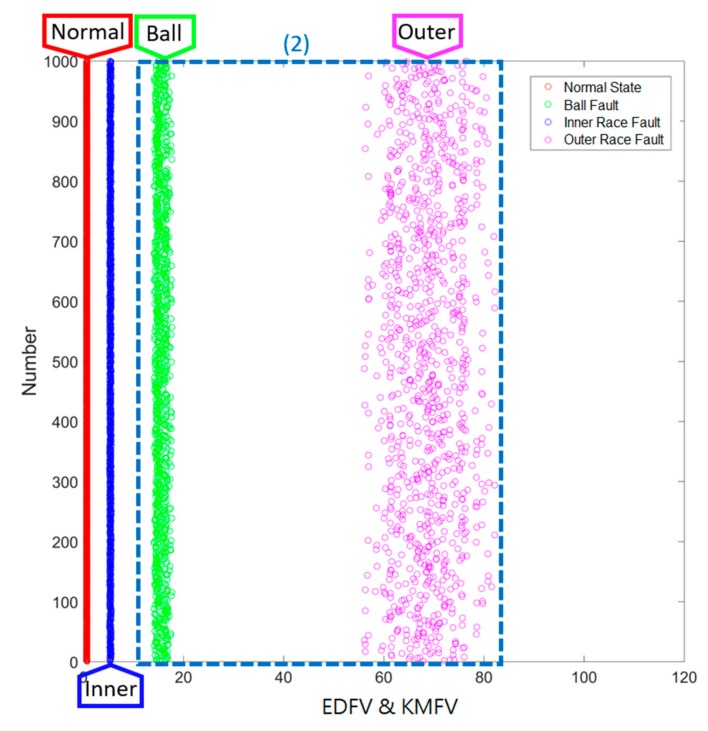
Results of Method 2: EDFV + KMFV (Hierarchical Structure).

The testing progress is the same as in Section 3.1, the last one-third of the data in the testing set is randomly picked up 1000 times in each of the different states, and the corresponding key feature values are accounted in real-time via the designed smart machine. The testing results are given in Figure 13, the horizontal axis refers to the range of key feature values, and the vertical axis reveals the testing time, 1000, for each of different states. It is very clear that the proposed Method 2 provides a more efficient and feasible way to detect different fault states.

### 3.3. Results of Smart Fault Detection Machine—Method 3: KMFV

**Figure 14:**
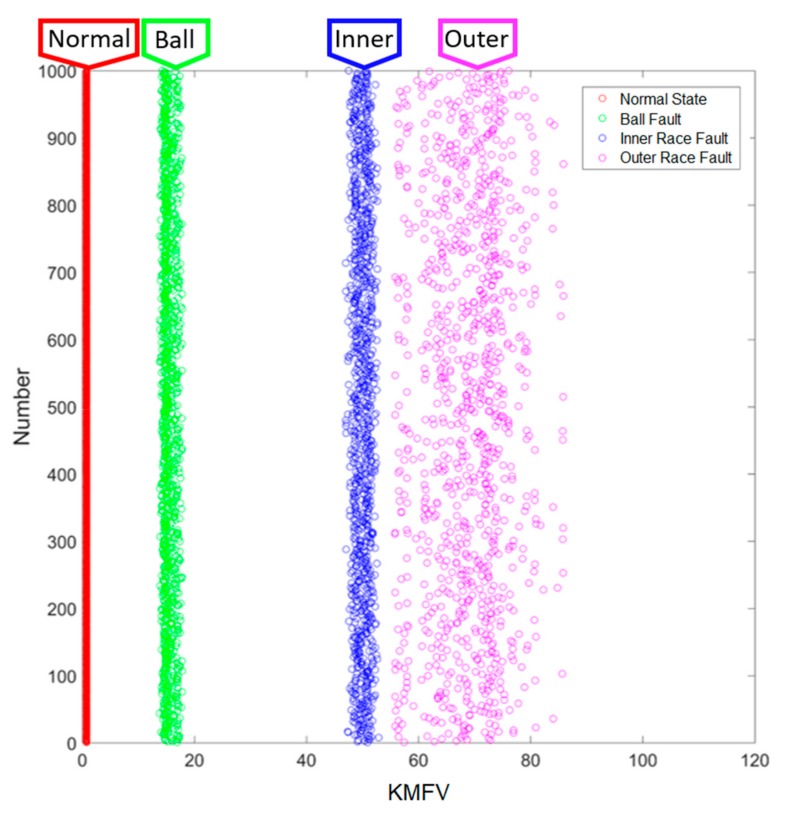
Smart Fault Detection Method 3.

For testing Method 3, only the range of KMFV is applied, where the last one-third of the data in the testing set are randomly picked up 1000 times in each of the different states, and the corresponding key feature values are accounted in real-time via the designed smart machine. The testing results are given in Figure 14; the horizontal axis refers to the range of key feature values, and the vertical axis reveals the testing time, 1000, for each of the different states. In Figure 14, the normal state and the ball fault can be distinguished simply; however, the ranges of KMFV in the inner race fault state and the outer race fault state are very close to each other. Nonetheless, the experimental results show evidence that each of them can be grouped together without any overlapping.

Comparing the experimental results of the proposed three methods, all three methods are effective, and the testing results show strong evidence for a high accuracy rate of identification. Moreover, according to the experimental results, in this study, the proposed Method 2 with hierarchical structure is better recommended to be able to provide a more robust, effective, and feasible real-time monitoring smart machine for fault-detection of a ball-bearing system. For more information about the computational cost, in this study, a computer with the specification, Model: ASUS ESC500 G4, OS: Windows 10 Enterprise 64-bit, CPU: Intel Core i7 7700 @ 3.60GHz, MB: ASUS P10S WS, RAM: DDR4 8GB 2400MHz, Graphics: Intel HD Graphics 630, Storage: Seagate BarraCuda 1TB (ST1000DM010), is applied to further train and test the smart machine, and the corresponding computational cost can be provided as follows in Table 5:

## 4. Conclusions

A smart fault-detection machine is proposed for an industrial ball-bearing system in this paper, where the original vibration signals are converted to the chaotic domain to further produce key features for each of the different states via the chaotic mapping strategy. Through experiment investigations, the key features produced via this smart machine can be applied to detect different fault states in ball-bearing system effectively. There are four main contributions of this paper, which can be summarized as follows: (1) 20,000 data are selected to be a training and testing unit (around 0.4 s), which leads the proposed machine to be uninfluenced by disturbance or error in the data collection stage. (2) Through developing the error dynamic system, and mapping those vibration signals into the chaotic domain, the unclear signals have been converted to easily distinguishable 3D phase portraits. (3) By constructing feature extraction approaches, the corresponding ranges of each state can be collected as well as adjusted until the definite-range of each state has been obtained. (4) The required computation resources are less, and the method developed is much simpler. One thousand testing units for each of the different states (equal to 80,000,000 data) are randomly picked up in the testing set to further investigate the performance of this smart fault-detection machine. The experiment results reveal that the proposed machine is effective and feasible to detect different kinds of fault states, and further, the performance and the accuracy are better than some traditional ways, for instance, Fourier Transform, Wavelet analysis, etc. The proposed smart fault-detection machine presents an efficient and simple strategy to real-time monitor states as well as for the safety of industrial systems.

## Figures and Tables

**Table 1:** Bearing fault state classification.

System Information	Conditions			
Sampling rate	48 (KHz)			
Fault diameter	7/14/21 (mil)			
Fault depth	0.011 (inch)			
Fault state	Normal/Ball/Inner race/Outer race			
Motor load	0 (HP)	1 (HP)	2 (HP)	3 (HP)
Motor speed	1797 (rpm)	1772 (rpm)	1750 (rpm)	1730 (rpm)
Motor speed	29.95 (rps)	29.53 (rps)	29.17 (rps)	29.83 (rps)
Period	0.0339 (s)	0.0337 (s)	0.0343 (s)	0.0347 (s)
Sampling points/1 period	1602 (points)	1625 (points)	1645 (points)	1664 (points)

**Table 2:** The detailed conditions of four stats in the considered ball-bearing system.

System Information	Conditions			
Sampling rate	48 (KHz)			
Motor load	0 (Hp)			
Motor speed	1797 (rpm)			
Period	0.0339 (s)			
Sampling points/1 period	1602 (points)			
Fault diameter	21 (mil)			
Fault depth	0.011 (inch)			
**States**	**Normal State**	**Ball Fault**	**Inner Race Fault**	**Outer Race Fault**
Total data	243,938	243,938	244,339	246,342

**Table 3:** The detailed operation procedure of the smart machine for different four states.

System Information	Normal State	Ball Fault	Inner Race Fault	Outer Race Fault
Total Data	243,938	243,938	244,339	246,342
Training Set	162,625	162.625	162,893	164,228
Testing Set	81,313	81,313	81,446	82,114
**Unit**	**20,000 (12.5 periods)**			
Training Time	1000 times			
Testing Time	1000 times			

**Table 4:** The ranges of feature values.

States	EDFV Range	KMFV Range
Normal State	0.69 < EDFV < 0.74	0.68 < KMFV < 0.77
Ball Fault	2.97 < EDFV < 3.83	13.24 < KMFV < 23.1
Inner Race Fault	5.09 < EDFV < 5.46	43.9 < KMFV < 51.6
Outer Race Fault	3.91 < EDFV < 4.69	58.9 < KMFV < 91.4

**Table 5:** Computational cost of four different states.

Computational Cost	Normal State	Ball Fault	Inner Race Fault	Outer Race Fault
Testing unit/1 time	0.007 s	0.007 s	0.007 s	0.007 s
Testing unit/1000 times	7 s	7 s	7 s	7 s
